# Supplementary material for: Toward Early and Objective Hand Osteoarthritis Detection by Using EMG during Grasps
Source: Sensors (Basel). 2023 Feb 22;23(5):2413. doi: 10.3390/s23052413 (PMC10006890; doi:10.3390/s23052413)
Supplement: Supplementary file 1 [file sensors-23-02413-s001.zip › sensors-2200925-supplementary.pdf]

SUPPLEMENTARY MATERIAL

Néstor J. Jarque-Bou *, Verónica Gracia-Ibáñez, Alba Roda-Sales, Vicente Bayarri-Porcar, Joaquín L. Sancho-Bru and Margarita Vergara

February 17, 2023

This document provides the supplementary material to support the manuscript entitled "Toward early and objective hand osteoarthritis detection by using EMG during grasps" submitted to SENSORS

Table S1. Statistics (average (SD)) of all EMG characteristics for each spot, grasp and group.

			Spot						
		**Group**	**WF_UD**	**WF_RD**	**DF**	**TM**	**FE**	**WE_UD**	**WE_RD**
**P2D**	**NZC**	M	969.2(108.0)	784.9 (100.8)	871.4 (109.1)	759.2 (73.2)	848.7 (109.1)	900.6 (80.7)	930.4 (115.0)
		W	942.3(90.4)	745.6 (121.4)	786.4 (63.8)	758.8 (106.2)	833.3 (99.5)	895.4 (81.2)	888.4 (87.1)
		HOA	891.9(137.4)	727.4 (120.7)	776.9 (168.9)	727.3 (158.3)	808.0 (118.0)	863.5 (113.0)	827.5 (127.9)
	**EWL**	M	976.9(355.4)	906.5 (284.4)	883.3 (139.3)	1562.3 (381.5)	1593.7 (421.0)	1487.7 (385.8)	1308.5 (208.2)
		W	1176.5(280.3)	682.8 (224.9)	887.7 (154.2)	1236.5 (461.7)	1491.8 (421.4)	1571.1 (295.2)	1422.6 (334.0)
		HOA	760.5(280.9)	691.7 (215.8)	843.6 (256.7)	909.0 (407.5)	1206.9 (420.3)	1050.4 (421.1)	978.0 (298.5)
	**EMAV**	M	0.33(0.11)	0.34 (0.10)	0.32 (0.07)	0.62 (0.16)	0.58 (0.13)	0.52 (0.12)	0.46 (0.08)
		W	0.41 (0.09)	0.28 (0.11)	0.33 (0.05)	0.48 (0.16)	0.55 (0.13)	0.55 (0.09)	0.50 (0.11)
		HOA	0.27 (0.10)	0.28 (0.08)	0.33 (0.12)	0.37 (0.16)	0.45 (0.14)	0.38 (0.14)	0.36 (0.10)
	**MA**	M	0.21 (0.11)	0.22 (0.10)	0.19 (0.07)	0.54 (0.21)	0.50 (0.17)	0.42 (0.14)	0.34 (0.09)
		W	0.28 (0.10)	0.16 (0.10)	0.21 (0.05)	0.37 (0.19)	0.45 (0.17)	0.46 (0.11)	0.40 (0.13)
		HOA	0.15 (0.08)	0.16 (0.07)	0.21 (0.12)	0.24 (0.15)	0.34 (0.15)	0.26 (0.14)	0.24 (0.09)

			Spot						
		**Group**	**WF_UD**	**WF_RD**	**DF**	**TM**	**FE**	**WE_UD**	**WE_RD**
**LP**	**NZC**	M	907.8 (69.0)	733.2 (88.8)	850.9 (89.5)	855.2 (111.4)	848.8 (88.4)	864.3 (73.5)	981.6 (94.3)
		W	941.0 (75.5)	751.8 (114.5)	851.3 (116.0)	792.5 (96.1)	799.2 (81.1)	870.8 (68.8)	917.0 (100.0)
		HOA	862.1 (151.3)	727.7 (125.9)	845.6 (206.2)	708.6 (173.6)	765.8 (116.6)	850.5 (163.2)	815.0 (138.9)
	**EWL**	M	937.7 (270.8)	923.0 (288.8)	902.5 (304.8)	1450.5 (415.8)	1532.9 (319.5)	1181.4 (337.8)	1723.0 (412.9)
		W	1184 (523.7)	625.9 (236.0)	901.8 (458.4)	1067.1 (400.0)	1302.5 (508.7)	1317.5 (281.3)	1775.8 (580.4)
		HOA	643.8 (217.3)	646.0 (172.9)	749.3 (256.1)	841.0 (280.4)	962.3 (303.2)	850.6 (331.7)	1113.6 (393.6)
	**EMAV**	M	0.33 (0.09)	0.37 (0.11)	0.33 (0.12)	0.52 (0.14)	0.56 (0.11)	0.42 (0.11)	0.57 (0.12)
		W	0.40 (0.17)	0.25 (0.10)	0.33 (0.15)	0.40 (0.14)	0.49 (0.17)	0.47 (0.09)	0.61 (0.18)
		HOA	0.23 (0.07)	0.26 (0.07)	0.29 (0.14)	0.36 (0.14)	0.38 (0.12)	0.31 (0.11)	0.42 (0.15)
	**MA**	M	0.21 (0.09)	0.25 (0.12)	0.22 (0.13)	0.42 (0.17)	0.46 (0.15)	0.30 (0.12)	0.48 (0.16)
		W	0.29 (0.21)	0.14 (0.08)	0.22 (0.15)	0.28 (0.14)	0.39 (0.20)	0.36 (0.11)	0.55 (0.24)
		HOA	0.12 (0.06)	0.14 (0.06)	0.17 (0.14)	0.21 (0.12)	0.24 (0.12)	0.18 (0.10)	0.30 (0.17)

			Spot						
		**Group**	**WF_UD**	**WF_RD**	**DF**	**TM**	**FE**	**WE_UD**	**WE_RD**
**CYL**	**NZC**	M	747.7 (101.9)	612.2 (86.2)	588.9 (122.4)	651.3 (122.6)	695.0 (89.8)	888.4 (74.0)	828.2 (129.9)
		W	836.7 (118.9)	607.0 (63.8)	651.6 (147.3)	640.6 (116.5)	703.3 (95.8)	892.4 (90.7)	782.6 (91.9)
		HOA	722.6 (137.7)	601.6 (136.4)	652.3 (147.6)	670.7 (182.5)	738.8 (127.5)	797.0 (166.1)	725.5 (139.8)
	**EWL**	M	2120 (279.4)	1281.5 (193.4)	1793.6 (210.7)	1923.5 (306.4)	1589.3 (393.8)	1741.7 (375.2)	2031.8 (302.8)
		W	2084 (426.4)	928.0 (334.0)	1428.2 (330.7)	1363.1 (453.7)	1179.9 (257.7)	1723.2 (220.4)	1971.4 (337.4)
		HOA	1169 (364.9)	967.0 (326.2)	1246.6 (338.6)	923.7 (320.0)	1197.2 (377.9)	1235.2 (383.7)	1222.1 (385.6)
	**EMAV**	M	0.84 (0.13)	0.57 (0.09)	0.82 (0.10)	0.84 (0.15)	0.65 (0.17)	0.63 (0.14)	0.74 (0.07)
		W	0.77 (0.15)	0.41 (0.14)	0.62 (0.15)	0.59 (0.18)	0.48 (0.10)	0.61 (0.08)	0.75 (0.13)
		HOA	0.49 (0.16)	0.45 (0.15)	0.56 (0.15)	0.40 (0.12)	0.48 (0.14)	0.48 (0.14)	0.50 (0.14)
	**MA**	M	0.85 (0.20)	0.47 (0.11)	0.82 (0.15)	0.85 (0.22)	0.58 (0.23)	0.55 (0.19)	0.71 (0.12)
		W	0.76 (0.21)	0.29 (0.15)	0.54 (0.20)	0.50 (0.22)	0.37 (0.12)	0.53 (0.10)	0.73 (0.18)
		HOA	0.38 (0.18)	0.33 (0.17)	0.46 (0.18)	0.27 (0.12)	0.37 (0.15)	0.37 (0.15)	0.39 (0.16)

			Spot						
		**Group**	**WF_UD**	**WF_RD**	**DF**	**TM**	**FE**	**WE_UD**	**WE_RD**
**LUM**	**NZC**	M	931.9 (139.3)	713.0 (76.5)	639.6 (77.5)	715.2 (85.2)	806.7 (92.0)	913.8 (70.5)	880.8 (95.1)
		W	927.3 (92.8)	670.7 (78.2)	700.0 (114.0)	728.2 (116.5)	789.3 (54.2)	882.4 (84.3)	817.3 (82.3)
		HOA	787.7 (136.1)	656.1 (136.5)	652.8 (152.4)	675.0 (124.9)	748.0 (98.0)	799.5 (115.6)	774.6 (71.4)
	**EWL**	M	962.2 (305.5)	890.6 (206.0)	926.8 (147.6)	1198.9 (320.8)	1222.6 (275.1)	1202.8 (349.1)	1506.1 (393.6)
		W	1138 (386.4)	670.2 (220.4)	862.0 (322.5)	892.6 (300.5)	1123.7 (350.6)	1188.2 (242.9)	1513.3 (361.7)
		HOA	755.1 (235.0)	779.8 (281.7)	856.9 (232.4)	878.7 (344.3)	1189.4 (432.5)	957.9 (383.0)	1212.4 (353.1)
	**EMAV**	M	0.33 (0.08)	0.36 (0.08)	0.41 (0.07)	0.48 (0.11)	0.46 (0.10)	0.42 (0.11)	0.53 (0.12)
		W	0.40 (0.13)	0.28 (0.10)	0.35 (0.13)	0.36 (0.11)	0.43 (0.12)	0.42 (0.08)	0.56 (0.11)
		HOA	0.29 (0.08)	0.34 (0.12)	0.38 (0.12)	0.37 (0.14)	0.47 (0.16)	0.36 (0.12)	0.47 (0.13)
	**MA**	M	0.21 (0.08)	0.23 (0.09)	0.28 (0.08)	0.37 (0.13)	0.34 (0.11)	0.30 (0.12)	0.43 (0.14)
		W	0.28 (0.13)	0.16 (0.08)	0.24 (0.13)	0.23 (0.11)	0.31 (0.14)	0.30 (0.08)	0.46 (0.15)
		HOA	0.17 (0.07)	0.22 (0.12)	0.25 (0.12)	0.24 (0.15)	0.35 (0.18)	0.24 (0.12)	0.35 (0.14)

			Spot						
		**Group**	**WF_UD**	**WF_RD**	**DF**	**TM**	**FE**	**WE_UD**	**WE_RD**
**OBL**	**NZC**	M	803.7 (85.5)	640.9 (94.6)	630.0 (110.0)	628.9 (93.5)	738.3 (66.6)	914.9 (67.6)	861.2 (111.4)
		W	848.6 (101.1)	652.1 (93.8)	704.3 (175.1)	681.4 (147.5)	723.2 (94.8)	872.8 (84.2)	841.0 (97.1)
		HOA	734.6 (128.4)	621.2 (105.7)	677.9 (132.6)	687.4 (139.9)	765.3 (124.5)	775.8 (162.5)	768.4 (117.1)
	**EWL**	M	1779 (214.1)	1133.3 (267.4)	1601.9 (373.7)	1517.9 (205.1)	1472.9 (259.6)	1651.8 (334.9)	1808.9 (295.0)
		W	1675 (599.3)	720.9 (252.7)	1077.3 (419.1)	1052.3 (359.7)	1088.1 (402.4)	1499.1 (328.7)	1684.6 (445.7)
		HOA	1065 (349.3)	852.7 (272.6)	1119.0 (355.8)	910.7 (279.4)	1193.8 (387.1)	1187.8 (304.0)	1231.8 (328.2)
	**EMAV**	M	0.68 (0.07)	0.49 (0.09)	0.70 (0.12)	0.67 (0.10)	0.58 (0.11)	0.57 (0.11)	0.65 (0.07)
		W	0.61 (0.22)	0.31 (0.11)	0.45 (0.17)	0.45 (0.16)	0.43 (0.14)	0.53 (0.11)	0.62 (0.17)
		HOA	0.43 (0.15)	0.38 (0.13)	0.48 (0.16)	0.38 (0.11)	0.47 (0.14)	0.46 (0.11)	0.48 (0.13)
	**MA**	M	0.61 (0.10)	0.37 (0.11)	0.65 (0.18)	0.60 (0.14)	0.49 (0.14)	0.48 (0.14)	0.58 (0.09)
		W	0.56 (0.28)	0.19 (0.09)	0.34 (0.18)	0.34 (0.16)	0.32 (0.15)	0.44 (0.14)	0.56 (0.22)
		HOA	0.32 (0.17)	0.26 (0.14)	0.37 (0.19)	0.25 (0.12)	0.35 (0.15)	0.35 (0.13)	0.37 (0.15)

			Spot						
		**Group**	**WF_UD**	**WF_RD**	**DF**	**TM**	**FE**	**WE_UD**	**WE_RD**
**INTPP**	**NZC**	M	752.1 (134.1)	643.4 (110.0)	610.1 (110.1)	688.1 (107.6)	817.7 (84.6)	852.4 (88.0)	856.7 (118.0)
		W	844.8 (96.0)	634.8 (94.2)	678.4 (120.6)	664.5 (119.1)	769.2 (77.8)	873.5 (82.9)	827.4 (106.6)
		HOA	684.1 (115.9)	587.4 (97.2)	636.1 (94.6)	686.3 (125.2)	752.8 (101.0)	744.2 (153.5)	755.9 (80.8)
	**EWL**	M	1762 (334.2)	927.3 (203.6)	1383.9 (443.4)	1495.8 (237.4)	1527.6 (269.3)	1411.4 (288.6)	1622.3 (231.1)
		W	1743 (333.6)	671.0 (228.3)	1148.7 (261.4)	1040.1 (388.4)	1076.8 (309.8)	1377.4 (390.4)	1683.7 (519.1)
		HOA	1396 (424.0)	955.1 (350.0)	1177.5 (319.5)	964.3 (419.1)	1344.7 (420.1)	1261.1 (386.5)	1183.6 (308.5)
	**EMAV**	M	0.70 (0.19)	0.40 (0.09)	0.61 (0.19)	0.63 (0.13)	0.57 (0.08)	0.52 (0.11)	0.59 (0.10)
		W	0.65 (0.15)	0.29 (0.09)	0.49 (0.14)	0.44 (0.14)	0.41 (0.10)	0.49 (0.12)	0.62 (0.18)
		HOA	0.58 (0.15)	0.44 (0.15)	0.51 (0.14)	0.40 (0.16)	0.53 (0.15)	0.51 (0.16)	0.46 (0.12)
	**MA**	M	0.67 (0.27)	0.27 (0.10)	0.54 (0.26)	0.55 (0.18)	0.47 (0.11)	0.41 (0.13)	0.50 (0.14)
		W	0.60 (0.19)	0.17 (0.09)	0.38 (0.17)	0.32 (0.15)	0.29 (0.11)	0.39 (0.14)	0.57 (0.24)
		HOA	0.49 (0.18)	0.32 (0.17)	0.41 (0.16)	0.28 (0.18)	0.42 (0.18)	0.41 (0.18)	0.34 (0.14)

<u>SPOTS:</u> Wrist flexion and ulnar deviation (WF_UD); wrist flexion and radial deviation (WF_RD); digit flexion (DF); thumb extension and abduction/adduction (TM); finger extension (FE); wrist extension and ulnar deviation (WE_UD); wrist extension and radial deviation (WE_RD).

<u>GRASPS</u>: two-finger pad-to-pad pinch (P2D); cylindrical grasp (Cyl); lumbrical grasp (Lum); lateral pinch (LatP); oblique palmar grasp (Obl); intermediate power-precision grasp (IntPP).

<u>GROUP:</u> healthy men (M); healthy women (W); hand osteoarthritis subjects (HOA)
